# Risk Factors for In-hospital Mortality After Transarterial Intervention After Postpancreatectomy Hemorrhage

**DOI:** 10.1007/s00270-020-02509-2

**Published:** 2020-05-20

**Authors:** Steffen Wolk, Christoph Georg Radosa, Marius Distler, Hanns-Christoph Held, Jens-Peter Kühn, Jürgen Weitz, Thilo Welsch, Ralf-Thorsten Hoffmann

**Affiliations:** 1 Department of Visceral, Thoracic and Vascular Surgery, Medizinische Fakultät Carl Gustav Carus, Technische Universität Dresden, Dresden, Germany; 2 Institute for Diagnostic and Interventional Radiology, Medizinische Fakultät Carl Gustav Carus, Technische Universität Dresden, Fetscherstr. 74, 01307 Dresden, Germany

**Keywords:** Postpancreatectomy hemorrhage, Interventional treatment, Covered stents, Stent graft, Coils

## Abstract

**Purpose:**

Postpancreatectomy hemorrhage (PPH) is one of the leading causes of mortality after pancreatic resection. Late onset PPH is most often treated using a transarterial approach. The aim of this study was to analyze risk factors for in-hospital mortality after endovascular treatment.

**Methods:**

Between 2012 and 2017, patients who were treated endovascular due to PPH were identified from a retrospective analysis of a database. Risk factors for mortality were identified by univariate analysis.

**Results:**

In total**,** 52 of the 622 patients (8.4%) underwent endovascular treatment due to PPH. The primary technical success achieved was 90.4%. In 59.6% of patients, bleeding control was achieved by placing a stent graft and in 40.4% by coil embolization. The primary 30-day and 1-year patency of the placed covered stents was 89.3% and 71.4%, respectively. The 60-day mortality was 34.6%. The reintervention rate was higher after stent graft placement compared to coiling (39.3% vs. 21.1%, *P* = 0.012). In the univariate analysis the need for reintervention was associated with a higher in-hospital mortality (21.2% vs. 7.7%, *P* = 0.049). The use of an antiplatelet agent was associated with a decreased in-hospital mortality in the univariate (11.5% vs. 25%, *P* = 0.024) and multivariate analysis (HR 3.1, 95% CI 1.1-9, *P* = 0.034), but did not increase the risk of rebleeding.

**Conclusion:**

Endovascular management of delayed PPH has a high technical success rate. Stent graft placement showed a higher reintervention rate. The need for reintervention was associated with a higher in-hospital mortality but did not differ between coiling and stent graft placement.

## Introduction

Postpancreatectomy hemorrhage (PPH) is one of the leading causes of death after pancreatic surgery, with an associated mortality rate of up to 50% [1–4]. According to the definition of the International Study Group of Pancreatic Surgery (ISGPS), PPH can be classified as early (= / < 24 h after operation) and late (> 24 h after operation) onset [5]. Late onset PPH can occur from vessel erosion caused by anastomotic leakage, intraabdominal infection, or vascular injury during resection and pseudoaneurysm formation in the postoperative course [6].

Late onset PPH should be treated primarily by a transarterial approach in accordance with clinical standards. A meta-analysis concluded that early angiography and embolization or stenting is safe and should be the procedure of choice in this critical setting [7]. Surgery remains a therapeutic option if patients cannot be resuscitated for an interventional treatment [7]. The indication for using coils or stent grafts is differential. End arteries, artery stumps or arteries with an adequate collateral flow are mostly treated with coil embolization, whereas stent grafts were implanted in main arteries to preserve the vascular flow and avoid organ dysfunction. A few studies have also shown the evolution and increasing use of interventional treatment over time in the management of late onset PPH [4, 8].

Although interventional radiology is the method of choice in the treatment of late onset PPH from the splanchnic arteries in many centers worldwide, only a few small series (less than 30 patients) have reported the outcome of endovascular coil embolization or stent graft placement after PPH [9–11]. Nevertheless, PPH is still a challenging problem with a high in-hospital mortality.

Therefore, the primary aim of the present study was to analyze the outcome of endovascular treatment of late onset PPH in a larger cohort in order to determine risk factors for in-hospital mortality. Secondary outcome parameters were the incidence of postoperative pancreatic fistula (POPF), abscesses, need for relaparotomy and complications of the interventional treatment, e.g., postinterventional liver failure.

## Methods

### Data Collection and Study Population

All patients who underwent minimally invasive radiological intervention due to late onset PPH were retrospectively identified from a prospective pancreatic surgery database from September 2012 to June 2017. Clinical data, patient characteristics and histological diagnostic findings for each patient were collected from this database. All baseline patient characteristics are shown in Table 1.

**Table 1:** Patient baseline characteristics

Patient characteristics	n = 52
sex (m/w)	36/16
ASA score	
1	1
2	18
3	32
4	1
Celiac trunk stenosis	2
age (median + IQR)	68.5 (56.3–759
BMI (median + IQR)	26.4 (23.1–29)
Diabetes	12
Heart failure	4
Coronary heart disease	6
Arterial hypertension	35
Smoker	7
Chronic obstructive pulmonary disease	2

### Interventional Procedures

In patients with suspected bleeding, a triphasic (non-contrast, arterial and portal phases) computed tomography (CT) scan was performed whenever possible. If arterial erosion was confirmed (either by active bleeding, pseudoaneurysm or irregular arterial wall), patients were treated by an interventional radiologist under local anesthesia with anesthesiological standby. Percutaneous transfemoral or transbrachial access was used depending on the desired treatment (coil embolization or stent graft) as well as the angle between the visceral arteries and aorta. After a 4 French sheath was introduced using the Seldinger technique into the femoral or brachial artery, the abdominal aorta was catheterized in order to perform an aortography to localize the bleeding site, if the origin was not clearly identified before in the CT angiography. If no bleeding was located, selective angiograms of the superior mesenteric artery and the coeliac trunk were performed. Depending on the location of bleeding, coil embolization or stent graft implantation were the preferred techniques for endovascular treatment. The choice of treatment was at the discretion of the interventional radiologist. In general, peripheral arteries, arterial stumps or arteries with an adequate collateral flow were treated with coil embolization, whereas stent grafts were implanted in main arteries such as the hepatic and proximal mesenteric artery to preserve the vascular flow and avoid organ dysfunction. We used two different types of coils, the AZUR® CX Peripheral Coil System (Terumo Medical Corporation, Somerset, NJ) or the Tornado® Embolization Coil (Cook incorporated, Bloomington, IN) and two different types of stent grafts, the VIABAHN® Endoprosthesis (W. L. Gore & Associates, Inc; Flagstaff, AZ) or the LIFESTREAM® Balloon Expandable Vascular Covered Stent (BD BARD Peripheral Vascular, Tempe, AZ). If the bleeding artery was treated with a stent graft, a long 5 to 8 French sheath (depending on the type and diameter of the stent graft) was introduced into the celiac trunk or the superior mesenteric artery and a 0.035″ stiff guidewire was placed in a peripheral branch of the bleeding artery. After determination of the adequate diameter and length in the preinterventional CT and/or the angiography the stent graft was placed into the vessel and released. If coil embolization was necessary the bleeding artery was catheterized using a 0.018″ guidewire. In arteries with collateral flow the microcatheter was placed distal to the bleeding location to perform a “frontdoor-backdoor” coil embolization and in peripheral arteries or arterial stumps coils were released via microcatheter close to the bleeding location.

After stent graft implantation at least one antiplatelet agent was started together with therapeutic anticoagulation with heparin. Patients were discharged with dual antiplatelet therapy for 6 weeks after discharge and then mono antiplatelet therapy with lifelong acetyl-salicylic acid. After coiling no specific anticoagulation protocol was followed. If no other indications for anticoagulation were given, thromboprophylaxis with low-molecular-weight heparin (LMWH) was continued and if the patient had received antiplatelet therapy prior to intervention it was maintained dependent on the bleeding risk. Besides anticoagulation, no other treatment-related medication was administered.

### Definitions

Primary technical success was defined as a stop of contrast leakage or the absence of contrast filling of pseudoaneurysms on the completion angiogram and if there was no need for a relaparotomy within the next 24 h because of hemorrhage [12, 13].

The primary 30-day and 1-year patency of the placed stent graft was defined as vessel patency with no need for repeat intervention to restore patency. Hepatic infarction was defined as a new wedge-shaped area(s) of hypoattenuation at the periphery on follow-up contrast-enhanced CT [14]. Percentage of liver ischemia was calculated after performing volumetric analysis of the liver and the ischemic liver parenchyma. Endovascular intervention related complications were assessed according to the standards for classification of complications of the Cardiovascular and Interventional Radiological Society of Europe (CIRSE). Major complications were defined as a serious adverse event (grades 3–6) [15].

The follow-up intervals were 3, 6 and 12 months after discharge from the hospital and then yearly thereafter.

### Statistical Analysis

The statistical analysis was performed using SPSS, version 21.0 (SPSS, Inc., Chicago, IL). All clinical and pathological characteristics were grouped to build categorical or nominal variables. The following variables were included in the univariate analysis: sex, ASA score, celiac trunk stenosis, type of surgery [e.g., pylorus-preserving pancreaticoduodenectomy (PPPD), cephalic pancreaticoduodenectomy (CPD), distal pancreatectomy (DP)], arterial resection, portal vein resection, multivisceral resection, diagnosis and histology, bleeding site, intervention technique and target vessel patency, the formation of a POPF or intraabdominal abscess, postinterventional liver ischemia, onset of PPH [> postoperative day (POD) 28], postinterventional anticoagulation for patients with stent graft implantation (no therapeutic vs. ≥ 1 antiplatelet drug), and the need and reason for relaparotomy. Continuous data are presented as median ± interquartile range (IQR) unless otherwise indicated. Univariate examination of the relationship between the groups was performed with a chi-squared (χ2) test. For testing independent predictors for poor in-hospital mortality, a Cox hazard model using in-hospital mortality as dependent variable with stepwise backward eliminations based on the likelihood ratios was employed. The following independent variables were included in the model: approach of treatment (coiling, stent graft deployment), POPF, need for reintervention, stent thrombosis, coronary artery disease, use of antiplatelet agents). Independent predictors were expressed as hazard ratio (HR) and 95% confidence interval (CI). A two-sided *p* value < 0.05 was considered statistically significant.

## Results

### Patient Cohort

During the study period, 622 patients underwent pancreatic resections in our department. We excluded patients with intraluminal gastrointestinal bleeding (n = 53, 8,5%) and with intraabdominal bleeding from non-splanchnic arteries (n = 21, 3,4%). Fifty-two of the cohort (8.4%) underwent endovascular interventional treatment of PPH originating from splanchnic arteries. The median age of the study population was 68.5 years (IQR 56.3–75 years) and the majority of the patients were male (69.2%). The American Society of Anesthesiologists (ASA) scores were 3, 2, and 1 or 4 in 61.5%, 34.6%, and 1.9% of the patients, respectively. Baseline patient characteristics are presented in Table 1. Most of the patients received a pylorus-preserving pancreatic head resection (53.8%), a distal pancreatectomy (21.2%) or a Whipple procedure (15.4%). Twenty-four percent of the patients had a simultaneous venous resection, and 7.7% had an arterial resection and reconstruction. A multivisceral resection was performed in 15.4% of the cases. The histopathological report revealed a cancer diagnosis, pancreatitis and other diagnosis in 78.8%, 11.5% and 9.7% of the patients, respectively (Table 2). The median follow-up period of the patients who were discharged from hospital after operation was 12.5 months (IQR 9.25–26.75 months).

**Table 2:** Surgical treatment and histopathological diagnosis

	n = 52
Surgical treatment	
PPPD	28
DP	11
cPD	8
TP	3
DPPHR	1
Enucleation	1
Multivisceral resection	8
Venous resection	12
Arterial resection	4
Histopathological diagnosis	
PDAC	15
CCC	9
CP	6
Ampullary cancer	6
NET	4
IPMN	2
metastasis	2
others	8
Malignancy	41

*cPD* classic pancreatoduodenectomy; *PPPD* pylorus-preserving pancreaticoduodenectomy; *DP* distal pancreatectomy; *TP* total pancreatectomy; *DPPHR* duodenum-preserving pancreatic head resection; *NET* neuroendocrine carcinoma; *PDAC* periductal adenocarcinoma; *CP* chronic pancreatitis; *CCC* cholangiocellular carcinoma; *IPMN* intraductal papillar mucineos neoplasia

### Bleeding Localization and Interventional Treatment

In 43 patients (83%), a CT was performed within 24 h before interventional treatment. The hepatic artery (n = 10, 19.2%) and gastroduodenal artery stump (GDAS) (n = 19, 36.5%) were the most common sites of PPH, followed by the splenic artery (n = 8, 15.4%), superior mesenteric artery (n = 7, 13.5%), celiac trunk (n = 6, 11.5%), dorsal pancreatic artery (n = 1, 1.9%), and the inferior pancreaticoduodenal artery (n = 1, 1.9%) (Table 3). The median day of onset of PPH was POD 18 (IQR POD 12–28).

**Table 3:** Interventional procedure and outcome

	n = 52	(%)
Bleeding localization		
Hepatic artery	10	19.2
Celiac trunk	6	11.5
Splenic artery	8	15.4
SMA	7	13.5
GDAS	19	36.5
DPA	1	1.9
IPA	1	1.9
Interventional procedure		
Coil	19	36.5
Stent graft	28	53.8
1-year primary patency of placed stents	21	75.0
Technical failure	5	9.6
Reintervention	15	28.8
Postinterventional anticoagulation* (n = 50)		
Low-dose heparin	4	8.0
ASA	2	4.0
ASA + clopidogrel	8	16.0
Thromboprophylaxis with LWMH	19	38.0
Therapeutic i.v. heparinisation (pTT > 50 s)	3	6.0
ASA + thromboprophylaxis	9	18.0
None	5	10.0
Clinical outcome		
POPF	36	69.2
Intraabdominal abscess	21	40.4
Postinterventional liver ischemia*	13	25.0
Need for relaparotomy	28	53.8
In-hospital mortality	26	50.0
Mortality follow-up	34	65.4
60-day mortality	18	

*ASA* acetyl-salicylic acid; *LMWH* low-molecular-weight heparin; *GDAS* gastroduodenal artery stump; *SMA* superior mesenteric artery; *DPA* dorsal pancreatic artery; *IPA* inferior pancreaticoduodenal artery; *POPF* postoperative pancreatic fistula

*confirmed by computed tomography examination

### Outcome of Endovascular Intervention

**Fig. 1:**
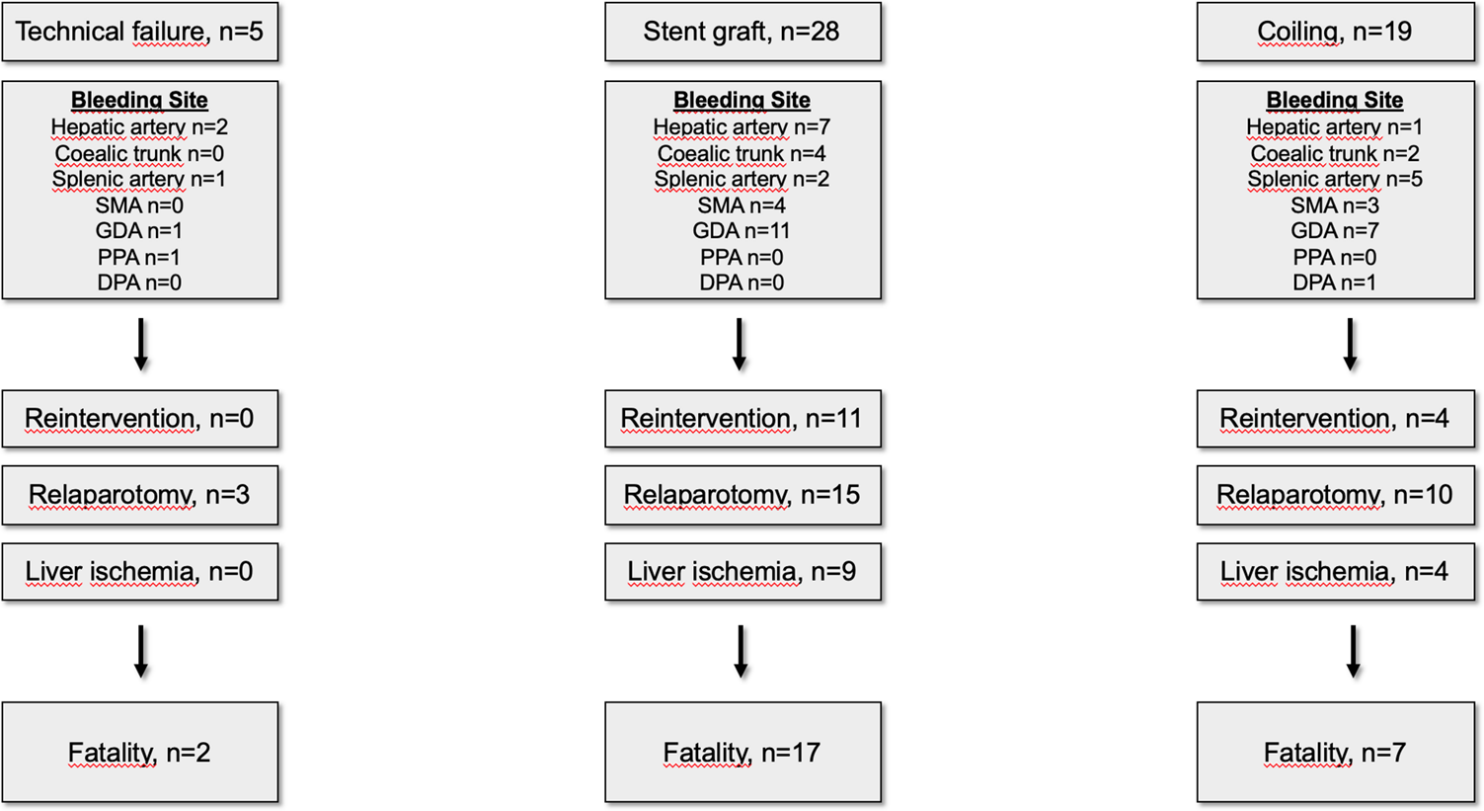
Flowchart after treatment allocation and postinterventional follow up

Primary technical success was achieved in 47 of 52 patients (90.4%) (Fig. 1). Of these 47 patients, 19 (40.4%) were treated with coil embolization and 28 (59.6%) with stent grafts. The median covered stent length was 37 mm (IQR 26–37 mm) and the median diameter was 5 mm (IQR 5–6 mm). In 5 of the 28 patients, 2 stents were placed in one session. In 2 cases (splenic artery stump, dorsal pancreatic artery), the endovascular treatment approach failed to stop the bleeding during angiography (Table 3). In case of the splenic artery stump, the stump was too short for coil embolization and stent graft placement was not possible due to a tortuous hepatic artery. For the dorsal pancreatic artery coil embolization was intended but because of the tortuous vessels the microcatheter could not place into the target artery. These two patients were managed by a watch-and-wait approach. Neither of them experienced a recurrence of hemorrhage during the clinical course. In 3 cases, the bleeding arteries (common hepatic artery or right branch of hepatic artery) could not be treated with stent grafts, therefore the interventional approach was abandoned and these patients then underwent a relaparotomy. In these cases, the site of bleeding was next to the ostium of important branches of the hepatic artery, which could otherwise be occluded. Bleeding control was achieved in 2 cases with total pancreatectomy and in 1 case the patient died because of persistent hemorrhage. The postinterventional anticoagulation protocol varied between the patients because of the interventional treatment methods (coils or stent grafts), different clinical courses, comorbidities, and the respective risk of the patients (Table 3). The primary 30-day and 1-year patency of the placed covered stents during the follow-up was 89.3% (n = 25 of 28 cases) and 71.4%. (n = 20 of 28 cases), respectively.

The endovascular reintervention rate was 28.8% (4 cases after coiling and 11 cases after stent grafts). The median day of reintervention after the first intervention was day 11 (IQR 3.5–25). The reasons for reintervention were recurrence of hemorrhage distal to the initially managed site (n = 8, 53.3%), catheter-directed thrombolysis of stent thrombosis (n = 3, 20.0%), diagnostic reasons (n = 2, 13.3%), spasmolysis (n = 1, 6.7%), and new onset stenosis of the target vessel (n = 1, 6.7%).

Intervention related complications were stent thrombosis (n = 7), coil dislocation (n = 1), stent graft dislocation (n = 1) and postpuncture pseudoaneurysm of the femoral artery (n = 1).

**Fig. 2:**
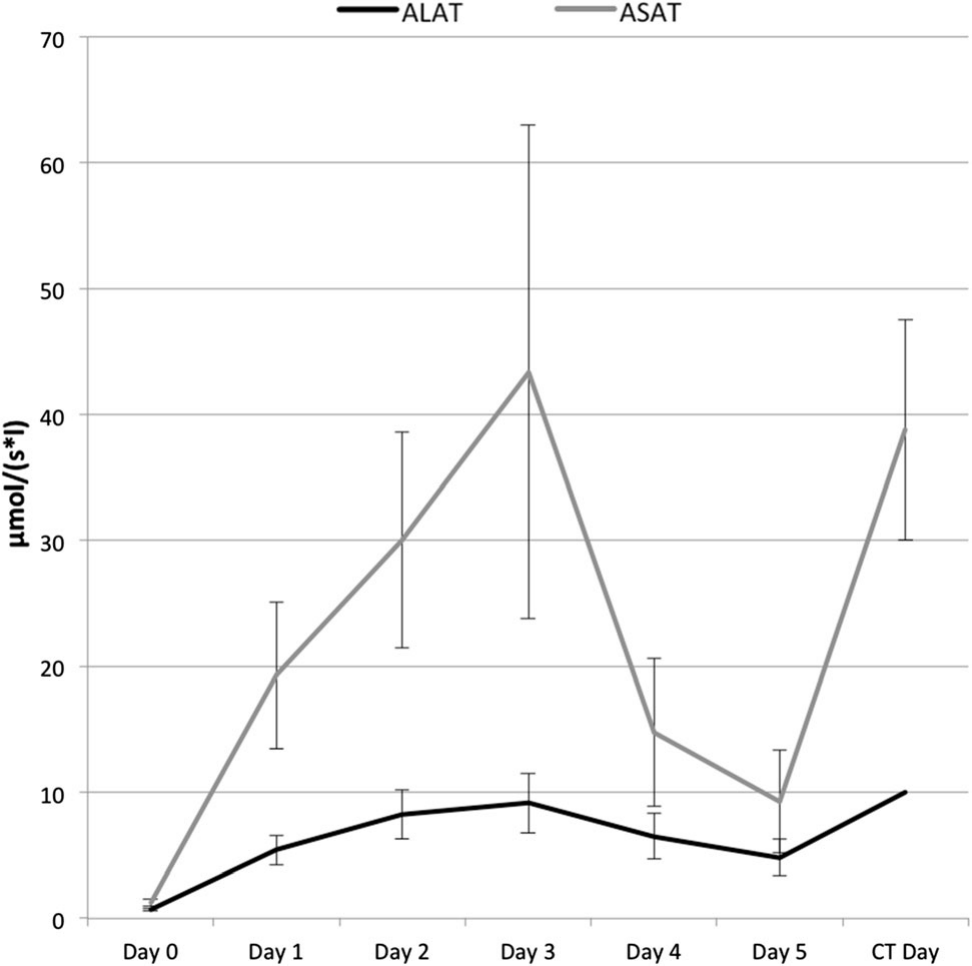
Postinterventional level of liver enzymes (ALAT/ASAT)

In addition to intervention related complications we observed surgery related complications. A concomitant POPF according to the definition of the International Study Group of Pancreatic Surgery (ISGPS) was diagnosed in 69.2% (n = 36) of the patients before hemorrhage and an intraabdominal abscess in 40.1%. In 30.8% (n = 16) of the patients, PPH was the first clinical sign of a POPF. Twenty-five percent (n = 13) of the patients had a postinterventional liver ischemia confirmed by a computed tomography (CT) examination. One patient developed a global ischemia with 100% ischemic liver parenchyma, five patients showed a segmental ischemia with 20% to 50% ischemic liver parenchyma and seven patients showed a segmental ischemia with under 20% ischemic liver parenchyma. The median postinterventional day of the CT examination was day 13 (IQR day 8–27). Figure 2 shows the postinterventional level of the liver enzymes aspartate aminotransferase (ALT) and alanine aminotransferase (AST). The serum AST enzyme level on postinterventional days 1 and 5 significantly correlated with the enzyme level on the day of the CT examination with evidence of liver ischemia (Pearson’s correlation coefficients 0.576, *P* = 0.04 on day 1; 0.69, *P* = 0.019 on day 5). Likewise, serum ALT enzyme levels on postinterventional days 1 and 3 significantly correlated with liver ischemia on the day of the respective control CT scans (Pearson’s correlation coefficients 0.716, *P* = 0.006 on day 1; 0.643, *P* = 0.024 on day 3).

After successful interventional treatment, 4 of 47 patients (8.5%) needed a relaparotomy due to persistent or recurrent hemorrhage during the follow-up. The bleeding site was confirmed in these patients by CT angiography and the origin was from non-splanchnic arteries (retroperitoneal, anastomosis). The overall relaparotomy rate due to hemorrhage was 19.2% (persistent hemorrhage n = 6, relaparotomy before intervention n = 3, recurrent hemorrhage n = 1). Surgical bleeding control was successfully achieved in 7 of the 10 cases with rehemorrhage. A relaparotomy due to other reasons was necessary in 34.6% of the 52 patients (removal of intraabdominal hematoma n = 8, anastomotic leakage n = 8, others n = 2). The median day of relaparotomy was POD 12.5 (IQR POD 10–24.75). The 60-day mortality of the cohort (n = 52) was 34.6% (n = 18) and the in-hospital mortality rate was 50% (Table 3).

### Risk Factors for In-hospital Mortality After Endovascular Treatment

Patient characteristics, type of operation, diagnosis, bleeding localization or kind of interventional procedure had no statistically significant influence on in-hospital mortality (Table 4). The univariate analysis showed that the necessity of endovascular reintervention was associated with an increased in-hospital mortality (21.2% vs. 7.7%, *P* = 0.049). Post/Periinterventional use of an antiplatelet agent (acetylsalicylate and/or clopidogrel; 31.5% after coiling and 50% after stent grafts) was associated with a decreased in-hospital mortality (11.5% vs. 25%, *P* = 0.024), which was confirmed by the multivariate analysis (HR 3.1, 95% CI 1.1–9, *P* = 0.034).

**Table 4:** Mortality analysis

	In-hospital mortality				* P* Value
	No		Yes		
	n	(%)	n	(%)	
Male	20	38.5	16	30.8	0.229
ASA score					
1	0	0.0	1	1.9	0.335
2	11	21.2	7	13.5	
3	14	26.9	18	34.6	
4	1	1.9	0	0.0	
Coeliac trunk stenosis	0	0.0	2	3.8	0.149
Operation					0.690
cPD	5	9.6	3	5.8	
PPPD	13	25.0	15	28.8	
DP	6	11.5	5	9.6	
TP	1	1.9	2	3.8	
DPPHR	0	0.0	1	1.9	
Enukleation	1	1.9	0	0.0	
Multivisceral resection	3	5.8	5	9.6	0.442
Arterial resection	1	1.9	3	5.8	0.298
Venous resection	6	12.0	6	12.0	1.000
Diagnosis					0.739
NET	3	5.8	1	1.9	
PDAC	7	13.5	8	15.4	
CP	3	5.8	3	5.8	
Ampullary cancer	2	3.8	4	7.7	
CCC	5	9.6	4	7.7	
IPMN	1	1.9	1	1.9	
Metastasis	2	3.8	0	0.0	
Others	3	5.8	5	9.6	
Malignancy	21	40.4	20	38.5	0.734
Bleeding localization					
Hepatic artery	4	7.7	6	11.5	0.797
Coeliac trunk	3	5.8	3	5.8	
Splenic artery	5	9.6	3	5.8	
SMA	3	5.8	4	7.7	
GDAS	10	19.2	9	17.3	
PPA	0	0.0	1	1.9	
DPA	1	1.9	0	0.0	
Hepatic artery or GDA	14	26.9	15	28.8	0.780
Interventional procedure					
Coil	12	23.1	7	13.5	0.246
Stent graft	11	21.2	17	32.7	0.246
Primary patency of placed stent grafts	9	33.3	11	40.7	0.148
Technical failure	3	5.8	2	3.8	0.246
Reintervention	4	7.7	11	21.2	0.049
Postinterventional antikoagulation (n=50)					
Low-dose heparin	1	2.0	3	6.0	0.124
ASA	1	2.0	1	2.0	
ASA+clopidogrel	5	10.0	3	6.0	
Thromboprophylaxis	8	16.0	11	22.0	
Therapeutic i.v. heparinisation (pTT > 50s)	2	4.0	1	2.0	
ASA+thromboprophylaxis withLMWHa	7	14.0	2	4.0	
None	0	0.0	5	10.0	
Paletet inhibitors	13	25.0	6	11.5	0.024
Clinical outcome					
POPF	19	36.5	17	32.7	0.548
Abscess	10	19.2	11	21.2	0.777
Postinterventional liver ischemia confirmed by CT examination	4	7.7	9	17.3	0.248
Relaparotomy	11	21.6	17	33.3	0.065
Hemorrhage	6	11.5	4	7.7	0.285
Removal of intraabdominal hematoma	2	3.8	6	11.5	
Anastomosis insuffciency	3	5.8	5	9.6	
Others	0	0.0	2	3.8	

*ASA* acetyl-salicylic acid; *LMWH* low-molecular-weight heparin; *GDAS* gastroduodenal artery stump; *SMA* superior mesenteric artery; *DPA* dorsal pancreatic artery; *IPA* inferior pancreaticoduodenal artery; *POPF* postoperative pancreatic fistula; *cPD* classic pancreatoduodenectomy; *PPPD* pylorus-preserving pancreaticoduodenectomy; *DP* distal pancreatectomy; *TP* total pancreatectomy; *DPPHR* duodenum-preserving pancreatic head resection; *NET* neuroendocrine carcinoma; *PDAC* periductal adenocarcinoma; *CP* chronic pancreatitis; *CCC* cholangiocellular carcinoma; *IPMN* intraductal papillar mucineos neoplasia

*confirmed by computed tomography examination

In 8 out of 19 patients with the use of an antiplatelet agent, it was there medication regime prior the intervention. The univariate analysis showed that patients with an antiplatelet agent had no higher rate of rebleeding (*P* = 0.585).

**Fig. 3:**
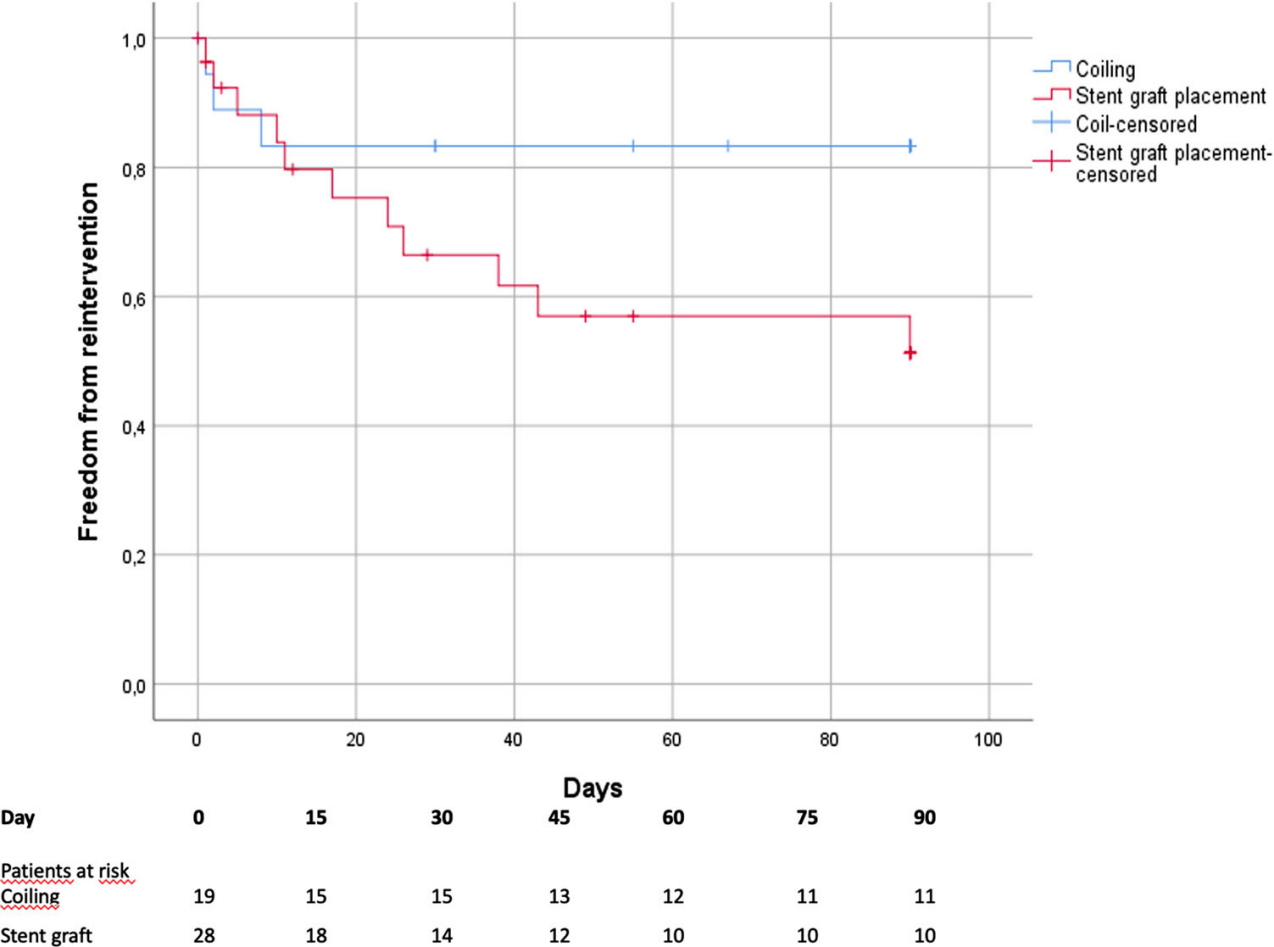
Freedom from reintervention

The reintervention rate was significantly higher after stent graft placement compared to coiling (n = 11, 39.3% vs. n = 4, 21.1%, *P* = 0.012). Figure 3 shows the freedom from reintervention after coil embolization and stent graft placement. There was no significant difference in mortality between patients without reintervention and patients with reintervention in the individual groups. The overall endovascular reintervention rate was 28.8%. The origin of bleeding was not associated with different reintervention rates in either the stent graft or coiling group. However, reinterventions were most frequent after bleeding from the gastroduodenal artery (n = 6 after stent graft, n = 2 after coiling). In addition, a relaparotomy during the postoperative course (before or after the first intervention) showed a significantly higher reintervention rate compared to patients without relaparotomy (23.5% vs. 3.9%, *P* = 0.013) (Table 5).

**Fig. 4:**
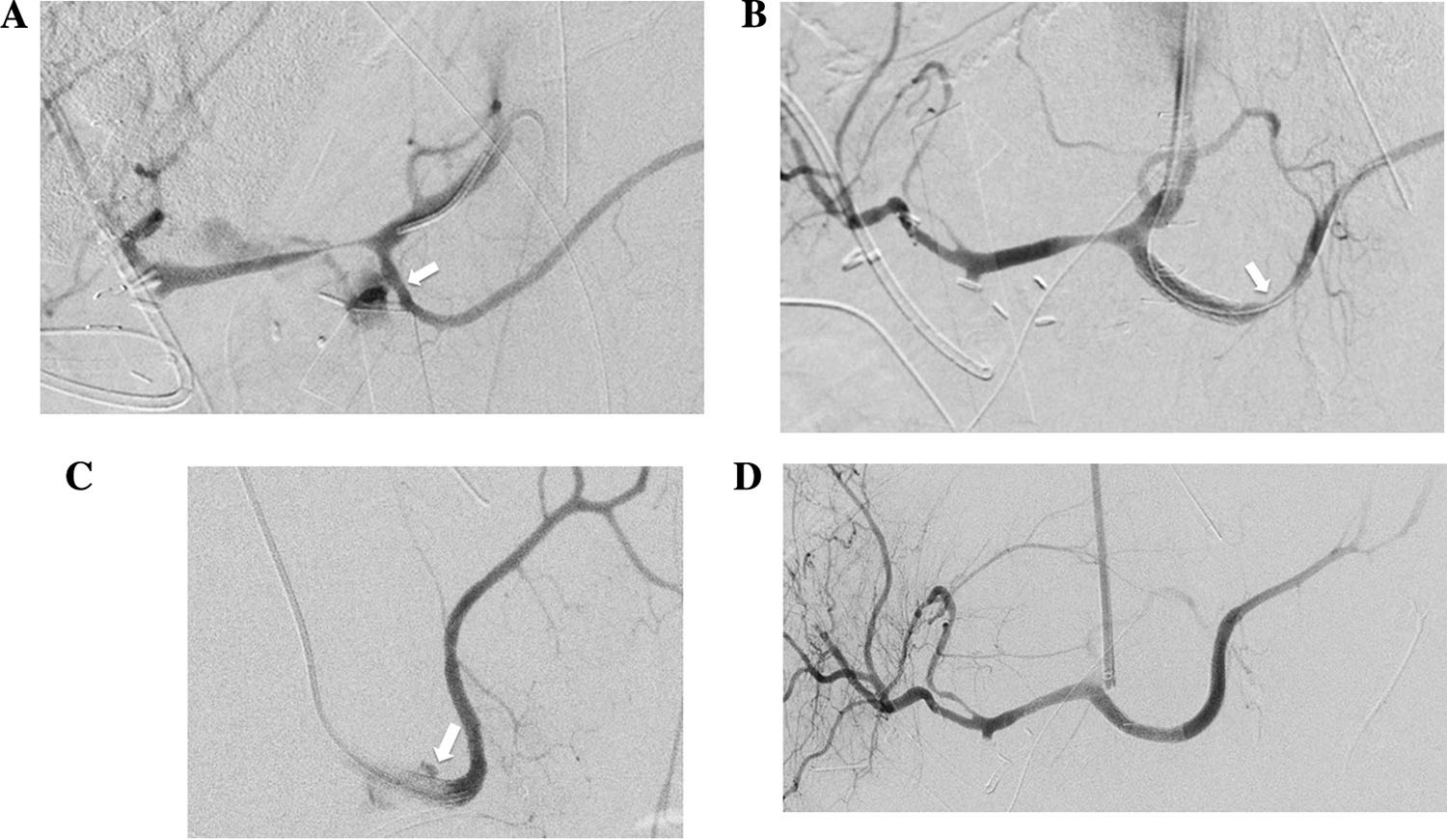
48-year-old male patient with pancreatic ductal adenocarcinoma treated with distal pancreatectomy. **A** Angiogram of the coeliac trunk demonstrates bleeding of the splenic artery (arrow). **B** Angiographic control after stent-graft implantation in the splenic artery shows no evidence of bleeding and minor spasm of the distal splenic artery (arrow). **C** Reintervention eight days later with recurrence of bleeding distal to the initial implanted stent-graft (arrow). **D** Angiography after implantation of a second stent-graft shows a successful treatment of the bleeding

**Table 5:** Reintervention analysis

	Reintervention				* P* Value
	No		Yes		
	n	(%)	n	(%)	
Treatment					
Stent graft	17	60.7	11	39.3	
Coiling	15	78.9	4	21.1	0.012
Mortality					
Stent graft	9	3.2	8	2.9	0.295
Coiling	4	2.1	3	1.6	0.075
Relaparotomy					
No	21	91.3	2	8.7	
Yes	15	53.6	13	46.4	0.013

## Discussion

PPH is a serious complication after pancreatic surgery. In a recent series of 1,450 pancreatectomies, it was associated with a high in-hospital mortality of 27.4% [4]. Moreover, endovascular management of PPH after pancreatic resection is a highly demanding procedure in critically ill patients. Therefore, the first intervention should be optimal and tailored to the individual patient to avoid further complications (e.g., rebleeding or stent thrombosis/end-organ failure). In this study, we observed a 50% in-hospital mortality after endovascular treatment of the 52 patients with PPH. There was no significant difference in mortality between the two treatment approaches (stent grafts vs. coils). When comparing the 50% mortality rate with the reported rates (ranging from 7.1% to 60% depending on the different definitions of the study groups), it seems that we are in the upper range [6, 10, 11, 13, 16–18]. However, the vast majority of these studies involved small patient groups (between 14 and 42), with better overall results reported for them as well. For example, Asai et al. reported an in-hospital mortality of only 13% in a cohort of 32 patients. A limitation of their study, however, was that it did not capture patients who underwent reoperation exclusively for bleeding control due to limited access to interventional angiography [19]. In this study, all patients with PPH could be treated by an endovascular approach because interventional therapies were available 24/7. This also included hemodynamically unstable patients despite this being the main reason for not performing an angiography [7]. A similar patient cohort reported by Miura et al. showed a slightly higher in-hospital mortality of 58.3% after endovascular treatment of hemorrhage following pancreaticobiliary surgery [3].

Despite the high mortality rate, hemostasis was achieved in 90.4% of the patients in this study. Therefore, recurrent hemorrhage was the reason for mortality in only 15.4% of patients (n = 8). In most cases, multiorgan failure (n = 15, 57.7%) led to death, followed by respiratory failure, primary disease and ischemic complications. Achieving hemostasis is therefore a critical step. However, the sequelae of an acute hemorrhage or a septic condition cannot be controlled in all cases.

Procedure-related complications after pancreatic surgery are common and can be managed by an interventional radiologist in most cases [20]. In a meta-analysis of 15 studies carried out by Roulin et al., interventional radiology was favored over relaparotomy in terms of mortality (22% vs. 47%; *P* = 0.02). However, complete hemostasis was achieved in virtually the same number of patients (76% vs. 80%; *P* = 0.35) [7]. In contrast to the 76% in the meta-analysis by Roulin et al., we were able to achieve an excellent complete hemostasis rate of more than 90%. Nevertheless, recurrent bleeding is common [11, 13, 21]. Ching et al. reported that 26.3% of the patients showed recurrent bleeding [13]. In this study, the overall angiographic reintervention rate was 28.8%. The reintervention rate was higher in the stent graft group than in the coil group with 39.3% and 21.1%, respectively. However, there was no significant difference in mortality between patients with reintervention and patients without reintervention in the individual groups.

The main reason for the difference in reintervention rate is mostly due to the fact that coiling and stent graft placement pursue different goals. Both techniques should achieve hemostasis but in contrast to coiling which causes an occlusion of an artery, stent graft placement should additionally preserve the vascular flow in main arteries to avoid organ dysfunction. Therefore, the maintenance of sufficient blood supply was a major reason for reintervention in the stent graft group with 33.4%. Another additional risk factor for reintervention after stent graft placement is the progressive erosion of the arterial wall distal to the initially covered artery caused by POPF or a surrounding abscess (Fig. 4). For this reason, hemorrhage recurred in 53.3% of our patients, although in individual cases it was indistinguishable if recurrence of hemorrhage were caused by progressive erosion or undersizing the stent graft. Undersizing tends to occur because choosing the right size for stent grafts could be difficult due to vasoconstriction of the arteries during bleeding. Another reason for the difference in reintervention rate could be due to vasoconstriction of the arteries during bleeding, which makes it difficult to select the right size for the stent grafts.

With 19.2% and 36.5%, the hepatic artery and gastroduodenal artery stump were the most common origin of bleeding. In order to preserve vascular flow to the liver, stent grafts had to be used in most cases. Hassold et al. reported a primary patency of the placed stent grafts in 16 patients at day 30 and 1 year of 84% and 42%, respectively [11]. We demonstrated a 30-day and 1-year primary patency of the placed covered stents in 28 patients of 89.3% and 75.0%, respectively, which is much higher compared to the above-cited study. In order to preserve the patency of stent grafts, antiplatelet agents have to be used. However, the risk of immediate and continued hemorrhage has to be balanced against the possibility of thrombosis. That is why no consistent recommendation exists regarding this issue [4, 13, 22, 23]. In this study, patients with an antiplatelet agent had a reduced mortality (*P* = 0.024). Therefore, it seems that although there is some risk of continued hemorrhage, at least 1 antiplatelet agent should be used in all patients after weighing contraindications. For patients after coil embolization, the standard use of antiplatelets is not necessary, but if it was part of their medication regime prior to intervention the use was continued. In this study there was no higher risk of rebleeding for patients with antiplatelet therapy, but due to the small number of patients this should be further evaluated. Another complication is the occurrence of postinterventional liver ischemia. In this study, 9 out of 13 patients with liver ischemia died in the postoperative course, which is a rate of more than 70%. Only one patient developed a global ischemia with 100% ischemic liver parenchyma, the other patients showed a segmental ischemia with less than 50% ischemic liver parenchyma. Even if it was not statistically significant due to the small number of patients, other study groups have also reported an increased case fatality of up to 48% due to liver failure after hepatic ischemic injury [3, 24]. We showed that the level of liver enzymes in the early postinterventional course correlated with liver ischemia at a later time point, although the number of cases for this observation is low. Early monitoring of the serum enzyme levels on the first 1–3 days after intervention may prompt further imaging studies.

In order to avoid liver ischemia, the endovascular approach had to be abandoned in 3 cases because the bleeding arteries could not be treated with stent grafts. In these cases, coil embolization with occlusion of the hepatic artery was technically feasible but would had caused subsequent liver ischemia/ failure. After relaparotomy, bleeding control was achieved in 2 cases while preserving vessel patency. Therefore, an interdisciplinary approach should be used when considering and discussing treatment options.

The present study has certain limitations, which should be considered when interpreting the results. The main one is its retrospective design. The sample size of 52 patients is still a small number of endovascular treatments of PPH. Another weakness of this study was that we did not take into account the different competence levels of the various interventional radiologists.

## Conclusion

PPH is a severe postoperative complication with high mortality rates. Interventional endovascular management as a primary approach for treatment of delayed PPH has an excellent technical success rate and is the preferred option for rescuing these critically ill patients. The need for reintervention was associated with a higher in-hospital mortality but did not differ between coiling and stent graft placement. Early AST/ALT monitoring after endovascular intervention on the first postinterventional day should guide further diagnostic imaging to avoid liver ischemia.
